# Is Stiff Person Syndrome Benefited by Physical Therapy Intervention? Summary of Case Reports

**DOI:** 10.1155/2019/5613680

**Published:** 2019-03-24

**Authors:** Anandh Vaiyapuri, Prashant Kashyap, Nivedita Kashyap, Hariraja Muthusamy, Radhakrishnan Unnikrishnan, Mazen Alqahtani

**Affiliations:** Department of Physical Therapy, College of Applied Medical Sciences, Majmaah University, Majmaah 11952, Saudi Arabia

## Abstract

It is interesting to be aware that there is no Randomized Clinical Trials (RCT) research article except a few case-study reports which have been reported about the physical therapy (PT) intervention for stiff person syndrome (SPS). This study was designed to determine the benefits of PT in cases with SPS through analysis of case reports, thereby to raise awareness among physical therapist about the most beneficial PT interventions for SPS. We executed acomputer-based search with a diagnosis of SPS who underwent PT and articles published only in English. We selected case-study reports because of nonavailability of RCT articles to review the complaints, deformities, contractures, precipitating factors, interventions, outcomes, results, disability, and benefits of PT management among SPS. We concluded that PT training is substantiated to be a necessary and beneficial intervention in rehabilitation of patients with SPS.

## 1. Introduction

Stiff person, as the name suggests, makes a person completely stiff like a log which includes axial and appendicular musculature of the body. In this condition the tone of the muscle increases tremendously and gives an appearance as rigidity. The precise prevalence of SPS is obscure, although the incidence is estimated at approximately 1 in one million individuals between both genders, but predominantly female [1]. SPS usually becomes apparent sometime between 30 and 60 years of age [2]. SPS is a rare disorder which was first described in the medical literature by medical practitioner Moersch and Woltman in 1956 as stiff-man syndrome. The primary pathophysiology in SPS is thought to be the antibodies to the glutamic acid decarboxylase (GAD) inhibiting the rate-limiting step of *γ*-aminobutyric acid (GABA) [3, 4]. Decreased inhibitory GABA is considered to be the cause of the stiffness and spasms. Around 70% of the patients who have been reported with SPS presented with the antibody “glutamic acid decarboxylase” which is considered a marker for SPS and is a strong indication of this disease. Patients suffering from SPS exhibit a wide range of associated problems like frequent progressive muscle spasms especially the axial parts like paraspinal and abdominal muscles, decreased range of motion in joints, difficulty in ambulation leading to disability, and impairment in activities of daily living (ADL), thus affecting the quality of life. In some cases, pain and muscle spasms extend to appendicular region too. Diverse pharmacological regimens have been administered for the management of pain and spasms, such as corticosteroids, intravenous immunoglobulin, plasmapheresis, benzodiazepines, and baclofen.

When reviewing the research articles that are published about SPS, many of them have no information regarding the interventions of PT for the patients with SPS. Very few researchers have recommended PT for patients with SPS [3, 5–8], but they have provided with little insight regarding specific PT management strategies.

George et al. [9] presented his first article that reported the PT intervention for SPS in 1984. Till now we could find only case reports which suggested about the pathology and its related medical and PT managements. There is no definite approach to this condition as the syndrome is in itself one of the rarest disorders. Only very scant case reports have stated that apart from pharmaceutical regimens for SPS, the physical therapy treatments can be applied for the management of pain, muscle stiffness, spasm, and functional disability.

Globally, debates are going on over the advantages of PT in the management of SPS. Although there are evidences that exercises and functional training may be useful, the exact effects of other forms of PT treatment either have not been studied or are disputed.

Thus, the purpose of this review is to dispense an overview of SPS and to document the cases describing the usefulness of PT interventions in a patient with disability due to SPS.

## 2. Method

**Figure 1:**
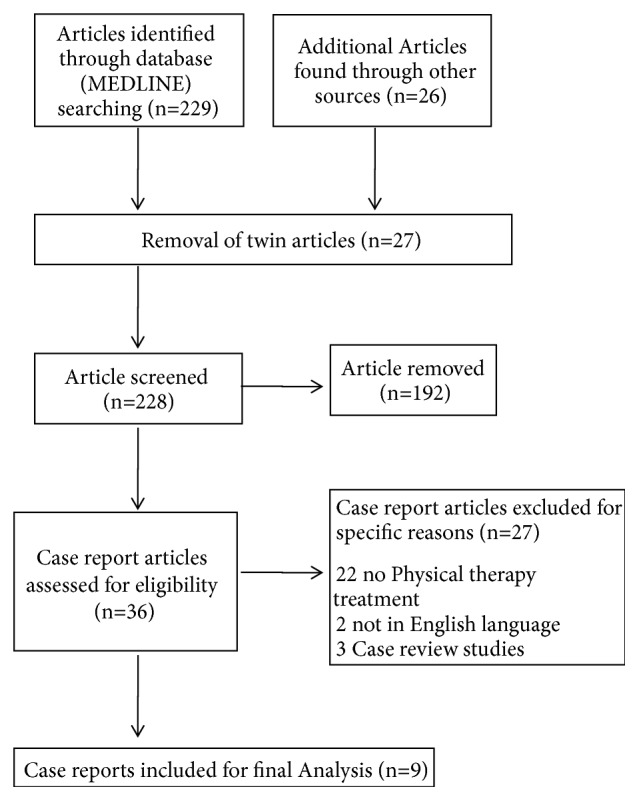
Flowchart representation of the study.

We executed a computer-based search for original relevant research articles consistent with a diagnosis of SPS underwent PT published only in English. We found no clinical trials except few case reports. The flow diagram of our study is shown in the Figure 1. Out of 228 articles identified in the database only 36 studies were case reports that were evaluated with a diagnosis of SPS. After evaluation, 27 case studies were excluded, of these 22 underwent only pharmacology treatment, 2 are articles not in English language, and 3 articles were review of case studies. So 9 case studies which underwent PT were included for analysis.

## 3. Summary of Case Reports Analysis

### 3.1. Personal Data Base and Patient Complaints of SPS

The patients who underwent PT treatment included in these nine cases had a mean age ranged from 30 to 60 years with few exceptions as it might be diagnosed early or late in the life [2]. The reports included patients of both sexes, but there was a predominance of females [1, 10]. Most of them were treated on an inpatient basis which might be due to the severity of the condition. There was limited or no information regarding their occupation or marital status. All the patients had complaints of severe pain and spasm leading to decreased range of motion, difficulty in ambulation, and inability to perform their activities of daily living as shown in Table 1.

### 3.2. Deformities, Contracture, and Precipitated Factors of SPS

All the case reports suggested that the condition affected the axial skeleton which includes the cervical lordosis, thoracic kyphosis, and lumbar lordosis. Few of them also presented with scoliotic deformities while others had contracture and deformities of hip, knee, and ankle musculatures and joints. There seems to be very less information regarding the precipitation factors except for a few who reported psychological stress as their aggravating factors as shown in Table 2.

### 3.3. Interventions of SPS

The most common PT intervention used in these case reports is massage, electrotherapeutic modalities, hydrotherapy, relaxation, and stretching. This treatment might be intended towards relieving spasm and emotional stress. Some patients were also intervened by ultrasound and soft tissue manipulation to relieve pain and spasm. Balance and coordination exercise, along with flexibility exercises, were also a part of the treatment. The intervention was also supported by the use of orthosis like leg casts, rolling walker, and ankle foot orthosis. The period of the intervention ranged from 2 weeks to 1 year. The frequency varied from once a week to daily sessions as shown in Table 3.

### 3.4. Outcomes and Results of SPS

The outcomes related to pain and spasm which were assessed by visual analog scale show significant decrease in pain and reduced spasm. Goniometric measurements confirmed the improvement in range of motion outcomes when assessed for joint ranges. Muscle strength outcome shows increase in the power after manual muscle testing was performed. Functional Independence Measure gave a clear picture of the flexibility outcome giving a marked improvement in their activities of daily living, thereby improving the quality of life as shown in Table 4.

## 4. Discussion

After studying all the case reports, it is evident that medical management no doubt provides complete treatment for the condition, but the main problem which has not been addressed satisfactorily is about the disability and its rehabilitation for SPS. The joint report formulated by World health organization (WHO) and World Bank states that disability will be an evolving global issue. The PT management plays a vital role in providing an optimal future for the various disabled patients [11]. These scientific literatures created an interest to review the effect of PT intervention among the SPS as these patients are disabled due to severe pain and muscle spasm. The review of 9 case studies revealed the severity of functional disability as 7 out of 9 cases were treated as in-patients with dependent ADL and reduced mobility. All these 9 cases reported pain, muscle spasm, and functional disability, thereby drawing attention to the effective management and rehabilitation of these patient complaints using appropriate PT interventions. The review of the case reports demarcated the PT interventions which were beneficial in minimizing pain, spasm, and improved functional mobility. The methods such as massage, stretching, range of motion exercises, and relaxation exercises were proved to be beneficial. Stretching exercises have a long history in improving the flexibility of muscles. The stretching exercises provided to the subjects with SPS showed satisfactory results by increased ROM and decrease in muscle spasm and the identical results were found in other studies [2, 6, 12, 13]. The stretching exercises combined with relaxation exercises displayed a beneficial improvement in the ADL function [6] and a marked decrease in severity and frequency of muscle spasm [14]. These reported results strongly support the stretching exercise to be placed on the top line management in the treatment protocol of SPS. Though the published studies failed to reveal the type of stretching exercise rendered, it is better to practice passive stretching exercise with the consideration of disability associated with SPS. The current review study strongly supports the stretching exercises as a medium to improve ROM among SPS. The beneficial effects are well explained by the fact that stretching exercise has the great possibility of increasing the number of sarcomeres in series caused by the stress generated in stretching exercises. The increased blood circulation caused by stretching exercise increases the viscoelasticity and reduces the stiffness of muscles and other connective tissues which results in improvement in joint ROM [15]. The relaxation exercise, massage, and soft tissue manipulation can be included in the PT protocol for SPS cases to minimize the disability. The cross-friction massage to the tendons reduced the muscle stiffness, thereby encouraging the patient to overcome their disability. The painful spasm in muscles was controlled by massage [12]. To avoid the development of long-term disability for the SPS patient the strengthening exercises should be included in the treatment protocol. The case study published by Karaoglan et al. proved the benefits of abdominal strengthening exercises in improving the Gait and lumbar movements. However, the strengthening exercises can be started in the form of isometric exercises to the major muscle groups of lower limbs as these exercises had direct effect on decreasing the severity of muscle spasm and pain [14]. There was a trend of better result recorded in the values of physiotherapy functional mobility profile scores when the treatment protocol included the balance and coordination exercise [13]. The patient showed good improvement when the posture correction exercises were included in the rehabilitation protocol. The literature evidence proved that these kinds of exercises help the patient to maintain an upright position and thereby control the fear of falls. To minimize the delirious effects of their disability, the rehabilitation strategies also included the use of orthotic devices like AFO, leg casts, and braces as suggested by Kelly et al. [16]. In all the available case reports none of them has mentioned about the effectiveness of electrotherapy modalities in the treatment protocol, except for Hegyi et al. who intervened using ultrasound to reduce the muscle spasm and joint stiffness. Apart from ultrasound modality, various forms of heat therapy and hydrotherapy (hot water) can be used to decrease the pain and to increase quality of life [17, 18]. The literature evidence proved that these kinds of exercises help the patient to maintain an upright position and thereby control the fear of falls. The review of case reports highlighted that there is a scarcity of available scientific evidences in planning the time duration for PT sessions to avoid fatigue among these SPS patients. The case report of Kahraman et al. 2016 followed 45 minutes of exercise sessions which was similar to the therapy session planned by Hegyi 2011. However, the entire treatment duration ranges from 10 days to 1 year and the reason for this wide disparity is due to the difference in clinical presentation from patient to patient. One among these case studies reported a strong recommendation to provide inpatient PT treatment services to SPS patient as it can efficiently address the disability needs of the patients [13]. The careful review of the available case reports strongly supports the PT interventions as a corner stone in rehabilitation of SPS; the physical therapy regime minimizes the disability and promotes a sense of independence among SPS.

### 4.1. Prognosis and Future Prospects

With SPS being so rare, a definite prognosis of the condition is still unknown. Though medical management has shown improvement in the severity of the syndrome, the frequency of getting recurrent and progressive spasms is still unclear. This severely impacts the patient's condition that tends to lose their flexibility, thereby ending with contractures, deformities, and impaired functional mobility. Progression of this condition can also increase the chances of mortality. PT intervention promises to have a major role in decreasing the decremental outcomes of the pain, spasm, and rigidity. This can be successfully achieved by including relaxation, massage, regular stretching, strengthening, and flexibility exercise in their rehabilitation protocol.

## 5. Conclusion

SPS is an uncommon practice among physical therapist. In this report, we describe patients with SPS in association with painful muscle spasm, tightness, contracture, postural abnormalities, and inability to ambulate accompanied by reduced ADLs. PT interventions in the form of massage, relaxation, ROM exercise, ultrasound, hydrotherapy, heat therapy, and stretching resulted in significant reduction of pain, spasm, and stiffness, thereby improving the joint ROM and making the individual less dependent for his ADLs. As an inpatient or outpatient, rehabilitation with early intervention of PT training was shown to be a crucial and imperative in the treatment of patients with stiff person syndrome. We concluded that PT training is substantiated to be a necessary and beneficial intervention in rehabilitation of patients with SPS.

## Figures and Tables

**Table 1:** Data base with patient complaints of Stiff man syndrome.

S.No	Study	Sex	Age	IP/OP	Occupation	Marital status	Patient Complaints
1	George et al. 1984	F	42	IP/OP	NI	Married	Could not walk and perform ADL without assistance Confined to bed Painful spasm in many muscles

2	Weissman et al. 2005	F	49	IP	NI	NI	Rigidity, Spasm and Contractures Inability to ambulate ↓ADL Painful back Muscle weakness Orthostatic hypotension

3	Potter 2006	M	33	IP	Laborer	Married	Pain spasm ↓ADL Need wheel chair for locomotion

4	Hegyi 2011	F	24	OP	Care giver	Single	Painful muscle spasm Gait anomalies ↓ROM in Lt LL

5	Kelly et al. 2014	F	64	IP	NI	NI	Pain stiffness with cognitive and functional decline

6	Koca et al. 2014	F	58	IP	NI	NI	Rigidity UL, LL and Abdominal muscle Painful muscle spasm Difficulty in walking

7	Karaoglan et al. 2015	F	26	IP	NI	NI	Anxiety LBP Abdominal and back muscles contracture Gait distraction

8	Kahraman et al. 2016	F	65	IP	NI	Married	Shoulder pain ↓Muscle strength Impaired balance in sitting ↓Functional mobility

9	Chang et al. 2016	F	55	NI	NI	NI	Painful muscle spasm in trunk and LL Intermittent stiffness in LL Difficulty in ambulation Several falls

F: Female; M: Male; IP: Inpatient; OP: Outpatient; Lt: Left; ↓: Reduced; UL: Upper Limb; LL: Lower Limb; ADLs: Activity of Daily Living; LBP: Low Back Pain; ROM: Range of Motion; NI: No Information.

**Table 2:** Deformities, contracture, and precipitated factors of stiff person syndrome.

S. No	Study	Deformities/contractures	Precipitated factors
1	George et al. 1984	Mild thoracic kyphoscoliosis B/L knees flexion contracture Rt heel cord shortening	Sudden noise Emotional stress

2	Weissman et al. 2005	NI	NI

3	Potter 2006	Hip flexion and plantar flexion with inversion of his ankle	Emotional stress physical exertion

4	Hegyi 2011	Moderate thoracic kyphosis Foot plantar flexed and inverted	NI

5	Kelly et al. 2014	B/L leg contractures	NI

6	Koca et al. 2014	Lumbar lordosis with elbow flexion	NI

7	Karaoglan et al. 2015	Minimal scoliosis Flattening of cervical lordosis and lumbar hyperlordosis	NI

8	Kahraman et al. 2016	Rounded shoulder and Forward head	NI

9	Chang et al. 2016	Hyperlordosis of lumbar spine Plantar flexed	Startle or Emotional upset

B/L: Bilateral; Rt: Right; NI: No Information.

**Table 3:** Intervention of stiff man syndrome.

S.No	Study	Intervention	Frequency/ wk	Duration	Supervision	Orthotics	HP
1	George et al. 1984	PT	NI	1 Month	NI	NI	NI

2	Weissman et al. 2005	PT	NI	2 Wks	NI	NI	NI

3	Potter 2006	FR Stretching Exs Relaxation Exs	Daily 2 Times/Wk	10 Days	Yes	RW	Yes

4	Hegyi 2011	US Soft tissue mobilization Stretching Exs	Once/Wk	15 Wks	NI	AFO	Yes

5	Kelly et al. 2014	NI	NI	1 Year	Yes	Leg casts and braces	Yes

6	Koca et al. 2014	ROM Exs Stretching Exs Relaxation Exs Isometric Exs MT	NI	NI	NI	B/L Elbow crutch	NI

7	Karaoglan et al. 2015	ROM Exs Stretching Exs Strengthening Exs	NI	NI	NI	Nil	Yes

8	Kahraman et al. 2016	Exs for Balance, Coordination, Posture, Strength, Flexibility FM Stretching Exs	5 Days/wk	1 Year	Yes	Walker	WA

9	Chang et al. 2016	PT	NI	NI	NI	NI	NI

PT: Physical Therapy; FR: Functional Retraining; US: Ultra Sound; Exs: Exercise; RW: Rolling Walker; AFO: Ankle Foot Orthosis; ROM: Range of Motion; B/L: Bilateral; FM: Functional Mobility; HP: Home Programme; WA: Ward Activities; MT: Mobilization Training; Wk: Week; NI: No Information.

**Table 4:** Outcomes and results of stiff man syndrome.

S.No	Study	Outcomes	Method of Assessment	Results
1	George et al. 1984	Rigidity in all joints LL ROM Painful spasm	NI	↓Stiffness ↓Rigidity ↓Pain ↓Cramps NN LL ROM
2	Weissman et al. 2005	Rigidity Spasm ADL	NI	↑Sitting ↓Spasm ↓Stiffness ↑ADL
3	Potter 2006	Ankle ROM Stiffness Spasm Pain Strength Posture Gait	Goniometer DTR VAS MMT SI TUG FIM Ruler	↑ROM ↑Posture ↑Gait ↓Pain ↓Spasm ↑ADL
4	Hegyi 2011	Pain ROM Strength Flexibility	VAS GM MMT 90-90 SLRT	↓Pain ↓Spasm ↑Gait ↑ROM ↑Flexibility
5	Kelly et al. 2014	NI	NI	↑ADL

6	Koca et al. 2014	NI	NI	↓Severity ↓Pain ↓ Spasm
7	Karaoglan et al. 2015	NI	NI	↓Pain ↓Stiffness ↑Gait ↑QoL
8	Kahraman et al. 2016	Pain Strength FM	NPS MMT PFMP	↑Balance, ↑Gait ↑FM
9	Chang et al. 2016	Strength Stiffness Sensitivity Spasm	MMT Stiffness index HSI mRS	↓Stiffness ↓Sensitivity ↓Spasm

ROM: Range of Motion; ADL: Activities of Daily Living; VAS: Visual Analog Scale; GM: Goniometer; SLRT: Straight Leg Raising Test; FIM: Functional Independence Measure; TUG: Timed Up and Go Test; MMT: Manual Muscle Testing; DTR: Deep Tendon Reflex; SI: Sensory Integrity; QoL: Quality of Life; NPS: Numerical Pan Scale; PFMP: Physiotherapy Functional Mobility Profile; FM: Functional Mobility; HSI: Heightened Sensitivity Index; mRS: Modified Rank Score; ↓: Reduced; ↑: Improved; LL: Lower Limb; NN: Near Normal; NI: No Information.

## Data Availability

The collected data used to support the findings of this study are included within the article.
